# Voltage and partial pressure dependent defect chemistry in (La,Sr)FeO_3–*δ*_ thin films investigated by chemical capacitance measurements

**DOI:** 10.1039/c7cp07845e

**Published:** 2018-04-05

**Authors:** Alexander Schmid, Ghislain M. Rupp, Jürgen Fleig

**Affiliations:** a Institute of Chemical Technologies and Analytics, Vienna University of Technology, Getreidemarkt 9 , Vienna , A-1060 , Austria . Email: alexander.e164.schmid@tuwien.ac.at

## Abstract

La_0.6_Sr_0.4_FeO_3–*δ*_ (LSF) thin films of different thickness were prepared by pulsed laser deposition on yttria stabilized zirconia (YSZ) and characterized by using three electrode impedance spectroscopy. Electrochemical film capacitance was analyzed in relation to oxygen partial pressure (0.25 mbar to 1 bar), DC polarization (0 m to –600 m) and temperature (500 to 650 °C). For most measurement parameters, the chemical bulk capacitance dominates the overall capacitive properties and the corresponding defect chemical state depends solely on the oxygen chemical potential inside the film, independent of atmospheric oxygen pressure and DC polarization. Thus, defect chemical properties (defect concentrations and defect formation enthalpies) could be deduced from such measurements. Comparison with LSF defect chemical bulk data from the literature showed good agreement for vacancy formation energies but suggested larger electronic defect concentrations in the films. From thickness-dependent measurements at lower oxygen chemical potentials, an additional capacitive contribution could be identified and attributed to the LSF|YSZ interface. Deviations from simple chemical capacitance models at high pressures are most probably due to defect interactions.

## Abstract 2

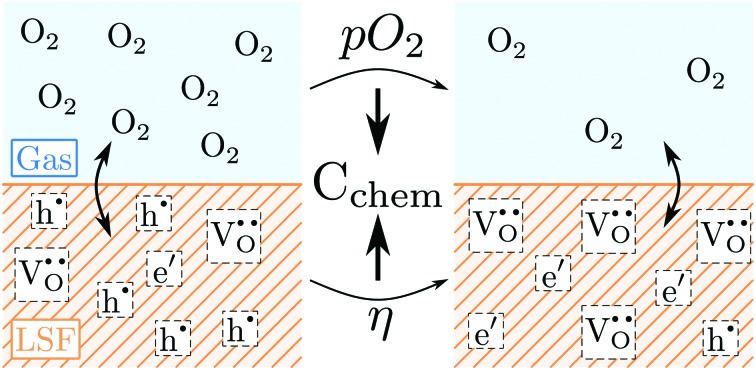
Chemical capacitance measurements are used to study the defect chemistry of La_0.6_Sr_0.4_FeO_3–*δ*_ thin films and their polarization (*η*) and *p*_O_2__ dependence. Important point defects are oxygen vacancies (
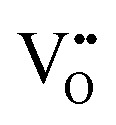
), electrons (e′) and holes (h˙).

## 1 Introduction

The perovskite-type oxide La_0.6_Sr_0.4_FeO_3–*δ*_ (LSF) belongs to the larger family of electrode materials with compositions of La_*x*_Sr_1–*x*_Co_*y*_Fe_1–*y*_O_3–*δ*_ (LSCF), which have been intensely studied as cathode materials in solid oxide fuels cells. Members of the LSCF family frequently exhibit low polarization resistances, especially in the Co rich regime. However, the Co-rich compositions also suffer from limited stability.^1^–^18^ The polarization resistance, and thereby the electrochemical performance of such electrodes, is strongly related to the ionic and electronic defects in the material. Not only are these defects necessary for the required mass and charge transport, they are also the reacting species involved in the electrochemical oxygen exchange reaction at the surface according to1
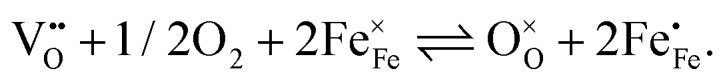
For a more detailed mechanistic understanding of this surface reaction, in-depth knowledge on the relation between polarization resistance and defect concentration is desirable, not only under equilibrium conditions but also under operating conditions, *i.e.* upon polarization.^19^

The equilibrium defect chemistry of LSCF has already been investigated on bulk samples by different methods, for example, coulometric titration, thermogravimetry, statistical thermodynamics calculations, carrier gas titration and electronic conductivity measurements.^5^,^20^–^28^ Much less data exist on the defect chemical relations upon electrochemical polarization, that is, under voltage bias.^5^,^10^,^11^ Moreover, the exact defect chemistry of LSCF thin films has hardly been investigated so far; thin film defect chemistry is not necessarily the same as for bulk materials, *e.g.* due to possible strain or interfacial effects. One experimental method particularly suited for investigating the defect chemistry of thin films is the measurement of the chemical capacitance, a capacitive property of mixed ionic and electronic conductors (MIECs) that depends on the charge carrier concentrations. This chemical capacitance reflects the ability of an oxide to change its stoichiometry in response to a change in oxygen chemical potential in the material.^29^ In the case of dilute defects and only one relevant electronic defect (eon), the chemical capacitance is given by^30^2
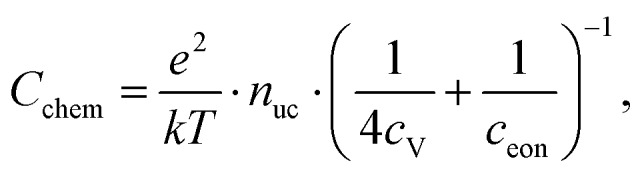
where *c*_V_ and *c*_eon_ denote the concentration (in defects per unit cell) of vacancies and electronic defects, respectively; *n*_uc_ is the concentration of unit cells and *e*, *k* and *T* have their usual meanings of elementary charge, Boltzmann constant and temperature, respectively.

This equation already shows the direct relation between charge carrier (*i.e.* defect) concentration and chemical capacitance. Detailed studies of chemical capacitances were performed on ceria thin films under reducing conditions.^31^–^34^ Some data exist on LSF in air and humidified hydrogen,^7^,^35^ on LSCF in air,^2^,^35^ and on La_0.6_Sr_0.4_CoO_3–*δ*_ in the range between 0.25 and 1 bar oxygen and under polarization.^5^,^12^,^35^ However, the chemical capacitance measurements of LSCF with in-depth quantitative analysis are still missing. More generally, chemical capacitance measurements are not yet an established routine tool for defect chemical investigations of thin films.

In this study, we use electrochemical impedance spectroscopy to investigate the defect chemistry of La_0.6_Sr_0.4_FeO_3–*δ*_ thin films *via* their chemical capacitance. A three-electrode approach allows variation of the electrode polarization (overpotential) and, together with variation of the oxygen partial pressure, defect chemical data become accessible over an oxygen chemical potential range spanning the equivalent of 15 orders of magnitude of oxygen partial pressure. Thickness dependent capacitance measurements reveal the existence of an interfacial capacitance in addition to the bulk chemical capacitance of the film. Defect formation enthalpies and entropies can be compared with those of dilute bulk defect models from the literature. Differences between measured thin film chemical capacitances and values predicted by literature data are discussed.

## 2 Experimental

### 2.1 Three-electrode samples

**Fig. 1:**
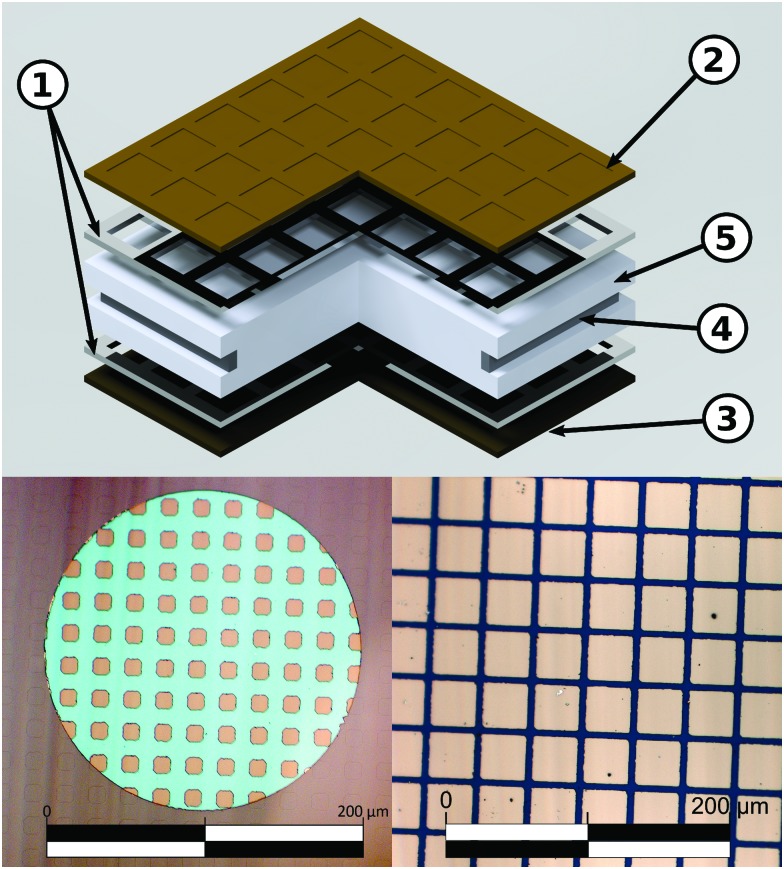
Top: Schematic of a three-electrode sample with platinum current collectors (1) beneath the LSF working electrode (2) and the counter electrode (3). A ring of porous LSF/Pt (4) around the circumference of YSZ (5) serves as the reference electrode. Bottom left: Bright field image showing a LSF thin film microelectrode. The platinum current collector grid is clearly visible through the electrode thin film. The active LSF surface area (directly above the electrolyte) is 25% of the total electrode area. Bottom right: Bright field image of a 3-electrode sample, the active LSF surface area is 73.5%.

Double side polished yttria stabilized zirconia (YSZ)(100) single crystals (Crystec) (5 × 5 × 1 mm^3^) were used as electrolyte substrates. A reference electrode was prepared by brushing a LSF/Pt-paste mixture into a notch around the substrate circumference and sintering for 2 h at 850 °C. Platinum current collector grids with 30/5 μm mesh/strip width and 100 nm thickness were prepared on both substrate sides by lift-off lithography and magnetron sputter deposition to ensure complete and homogeneous polarization of the entire oxide electrodes. Dense LSF thin film working electrodes were produced by pulsed laser deposition (PLD). The target was prepared from La_0.6_Sr_0.4_FeO_3–*δ*_ powder (Sigma Aldrich) by cold isostatic pressing (150 MPa) and sintering in air (12 h, 1200 °C). Phase purity of the target was checked by X-ray diffraction. Ablation was done at 600 °C substrate temperature and 0.04 mbar oxygen pressure using a KrF excimer laser (Complex Pro 201F, 248 nm) with 400 mJ laser pulses at 5 Hz. The target to substrate distance was 6 cm. Samples with LSF film thicknesses from 28 ± 5 to 116 ± 5 nm were produced; the film thickness was determined by profilometer measurements. Additionally, samples with platinum covered working electrodes were prepared by sputtering 300 nm of platinum onto LSF films of 28 ± 5 to 116 ± 5 nm thickness. Similar LSF films (using the same deposition parameters) were already described in ref. 7 and revealed columnar grain growth with a column size of about 50 nm. Porous La_0.6_Sr_0.4_CoO_3–*δ*_ counter electrodes with low polarization resistance were also prepared by PLD (400 mJ, 5 Hz) at 450 °C and 0.4 mbar oxygen.^36^ A sketch of the prepared three-electrode samples is shown in Fig. 1.

### 2.2 Microelectrodes

Further samples were fabricated with circular LSF microelectrodes of 200 μm diameter and 40 nm thickness. In this case, counter electrodes of porous LSF/Pt were brushed on the non-polished side of single side polished YSZ(100) single crystals and sintered for 2 h at 850 °C. Platinum current collector grids of 10/10 mesh/strip width were prepared by magnetron sputtering on the polished side, as described above. LSF thin films were again deposited on top by PLD. Microstructuring of the electrode films including current collector grids was done by argon ion beam etching (tectra GmbH, ionEtch Sputter Gun) at 1.1 × 10^–4^ mbar Ar, with a beam current of 2 mA for 25 min.

### 2.3 Impedance spectroscopy

Impedance spectra were recorded on a Novocontrol PotGal electrochemical test station and a Novocontrol Alpha A impedance analyzer in potentiostat mode, with applied DC voltages between the working and reference electrode from 0 to –600 mV. An AC voltage of 10 mV rms and a frequency range of 1 MHz to 10 mHz were used. Each frequency point was measured for at least one second and one period. Measurements were performed between 500 °C and 650 °C with different oxygen/nitrogen mixtures (0.25 mbar to 1 bar O_2_) (Alphagaz, 99.995%) in a closed apparatus of fused silica. For the three-electrode samples, working and counter electrodes were contacted between two platinum sheets, the reference electrode was contacted with platinum thread. Microelectrode samples were contacted by using a platinum sheet (counter electrode) and a platinum needle (microelectrode).

## 3 Results and discussion

### 3.1 Impedance spectra and determination of the chemical capacitance

**Fig. 2:**
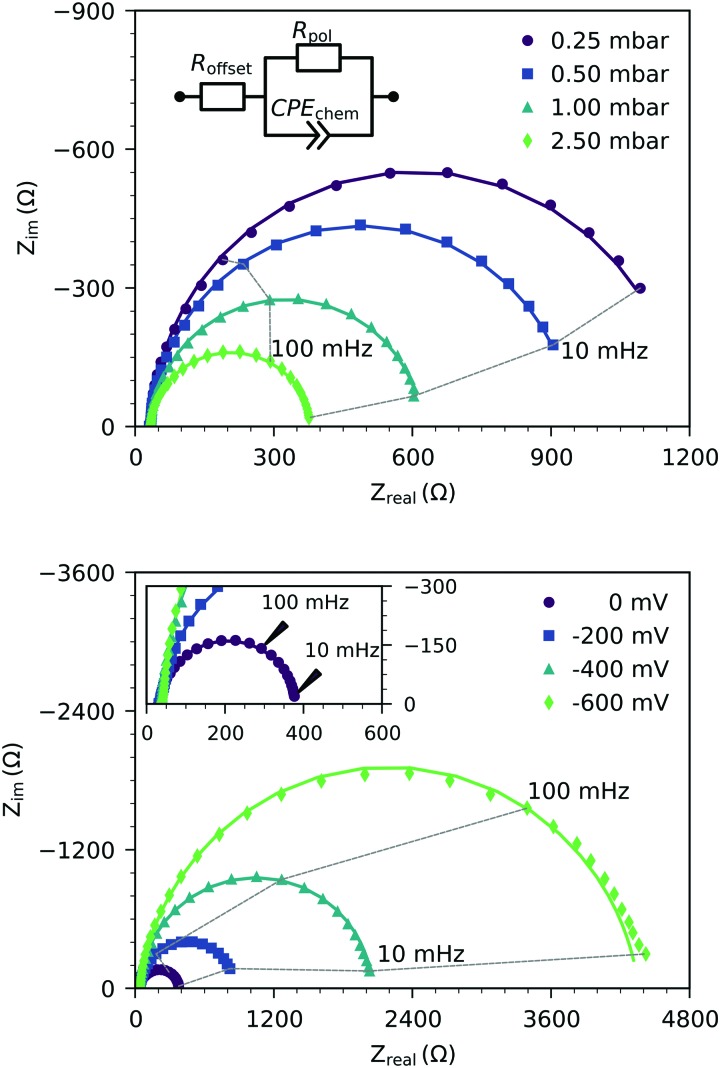
Typical impedance spectra of three-electrode samples measured at 600 °C under various oxygen partial pressures without bias (top), and under different cathodic polarization in 2.5 mbar oxygen (bottom). The spectra were determined on a 118 nm thick LSF film. Symbols are measured data, and lines represent fits of the low frequency data to the equivalent circuit model (see the inset). The inset in the bottom plot shows a zoom on the high frequency region.

Fig. 2 shows the representative impedance spectra for different oxygen partial pressures and DC bias voltages. The spectra exhibit a high frequency resistive offset and a dominant low frequency semicircle. The high frequency offset shows no dependence on oxygen partial pressure or applied DC voltage. The value of this high frequency offset (34 Ω at 600 °C) agrees very well with the ionic transport resistance in the YSZ electrolyte substrate between the working and reference electrode.

The low frequency semicircle shows a clear dependence on oxygen pressure and DC voltage. It can be fitted to a parallel R-CPE element and a series resistance, where the constant phase element (CPE) is used to model an imperfect capacitor with impedance *Z*_CPE_ = *Q*^–1^(*iω*)^–*P*^, see the circuit in Fig. 2.^37^ Experimental *P*-values close to one (typically between 0.91 and 1) indicate nearly ideal capacitive behavior. For certain measurement parameters, especially at low oxygen partial pressure, only part of the low frequency semicircle was accessible by the frequency range employed here. However, the capacitive contribution could always be determined from the measured data points with small fit errors (typically <1%). From the fit results (*R*_pol_, *Q*, *P*), the area-specific capacitance *C* was calculated by3
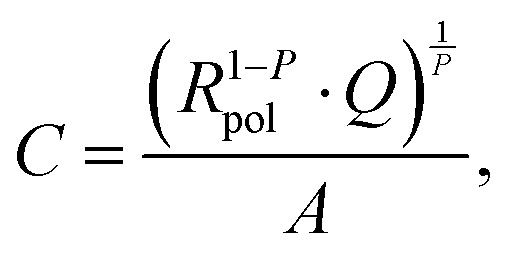
with the electrode area *A*.^37^ The resulting values in the range of 1 to 15 mF cm^–2^ are much higher than any double layer capacitances of an electrode, but they are very common for chemical capacitances found in LSCF electrodes.^2^,^7^,^10^,^38^

Hence, the dominating semicircle feature of the spectrum is interpreted in accordance with similar spectra found in the literature for LSCF electrodes^2^,^3^,^7^,^35^ or similar mixed conducting electrodes.^5^,^14^,^31^,^39^ This means that the resistance of the large semicircle is caused by the oxygen surface exchange reaction and the capacitance reflects the chemical capacitance of the film. The ionic and electronic across-plane transport resistances, as well as the in-plane electronic transport resistance, are negligible compared to the surface exchange resistance. Contributions from the LSF/YSZ interface can also be neglected, as the measured impedance spectra do not show any distinct interfacial (intermediate frequency) features. Moreover, from the literature and our own previous experiments with different current collector geometries, we can conclude that the electrode volume above the current collector grid is inactive towards oxygen exchange and does not contribute to the chemical capacitance due to the large in-plane ionic sheet resistance.^7^ Therefore, the chemical capacitance was normalized to the free thin film volume (73.5% of the total film volume for three-electrode samples, and 25% for microelectrode samples), *i.e.* it was calculated according to4
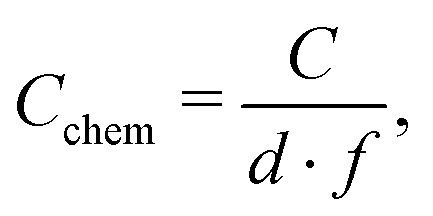
where *C* is the area specific capacitance, *d* is the film thickness and *f* is the active electrode fraction. It is noteworthy that while a continuous increase of the surface exchange resistance over the course of the experiment was observed, the chemical capacitance was very stable, with typically less than 1% change within 12 h of measuring time.

### 3.2 Oxygen partial pressure and voltage dependency of the chemical capacitance

**Fig. 3:**
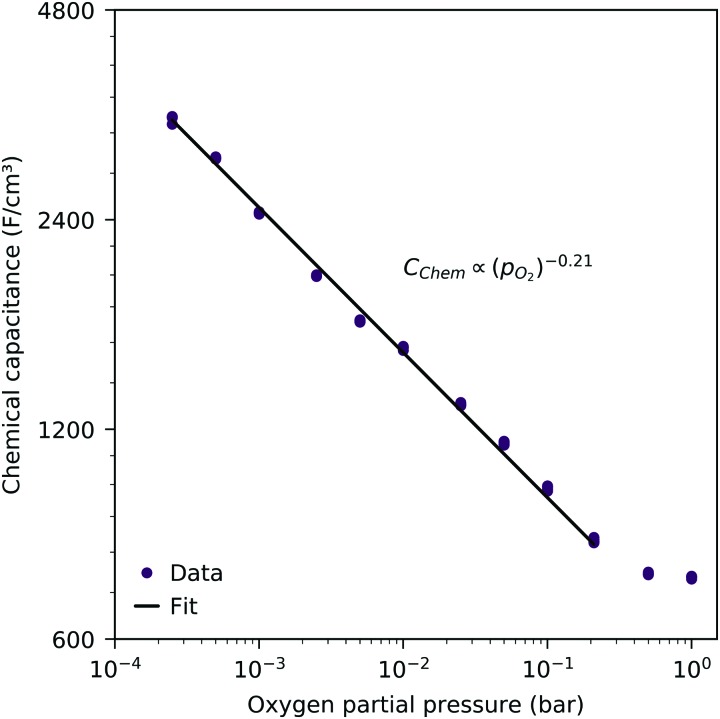
Equilibrium chemical capacitance at 600 °C (circles) as a function of oxygen partial pressure, measured on a 40 nm thin LSF film, and fit to a power law (solid line).

Fig. 3 shows the equilibrium chemical capacitance (*i.e.* without applied DC voltage) as a function of the oxygen partial pressure. With increasing oxygen partial pressure, the chemical capacitance decreases, see also the more detailed interpretation below. In the high *p*_O_2__ region, the decrease of the chemical capacitance levels off.

**Fig. 4:**
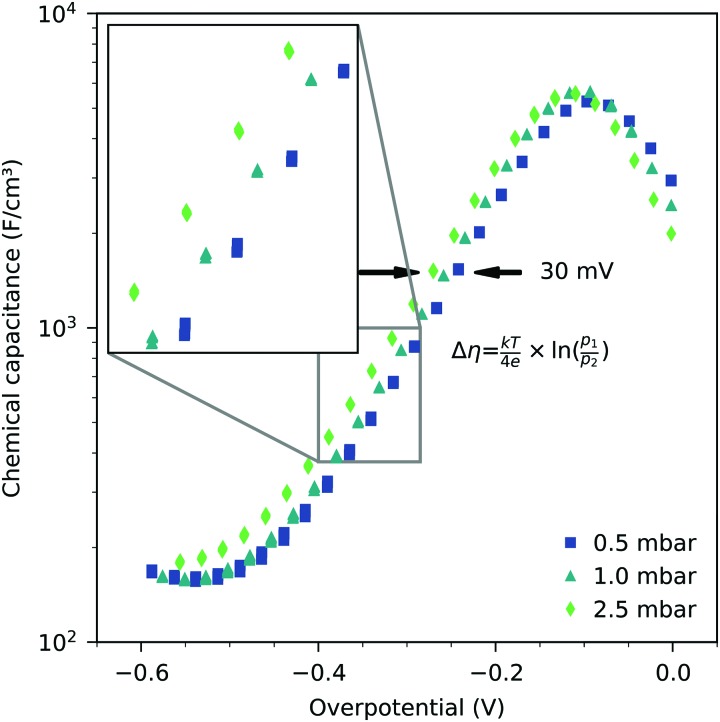
Chemical capacitance of a 40 nm thin LSF film, measured at 600 °C as a function of electrode overpotential and oxygen partial pressure. The shift on the potential axis is equal to the Nernst voltage between the atmospheres with oxygen partial pressures *p*_1_ and *p*_2_, respectively.

Fig. 4 displays the chemical capacitances under DC polarization, where the electrode overpotential *η* was calculated from the voltage between the working and reference electrode *U*_DC_ by correcting for ohmic losses in the electrolyte (*R*_offset_·*I*_DC_), *i.e.*

5 *η* = *U*_DC_ – *R*_offset_·*I*_DC_.

The ohmic resistance *R*_offset_ was determined by impedance spectroscopy, and *I*_DC_ is the measured current through the cell.

The *C*_chem_*vs. η* curves measured in different atmospheres are very similar except for a shift on the overpotential axis. This voltage shift Δ*η* between curves for different partial pressures (*p*_1_ and *p*_2_) equals the Nernst voltage calculated from these partial pressures according to6
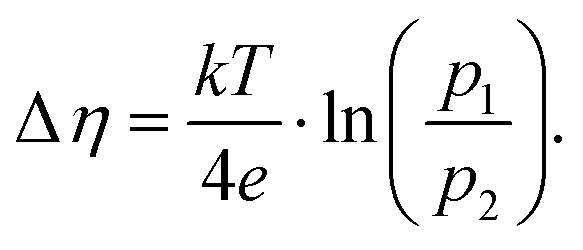



To further quantify and compare the impacts of oxygen partial pressure and electrode polarization, we have to consider the distribution of the oxygen chemical potential in a three-electrode system. We define an oxygen chemical potential of the surrounding atmosphere, which is assumed to be constant in the entire apparatus, *i.e.* at all electrodes. Pure oxygen at 1 bar is used as a reference for the oxygen chemical potential, *i.e. μ*1barO = 0. Without applied DC bias, all electrodes (working electrode WE, counter electrode CE and reference electrode CE) are in equilibrium with the atmosphere, thus their oxygen chemical potentials (*μ*WEO, *μ*CEO and *μ*REO) are equal to *μ*atO. The oxygen chemical potential in any electrode relative to 1 bar oxygen is thus given by7
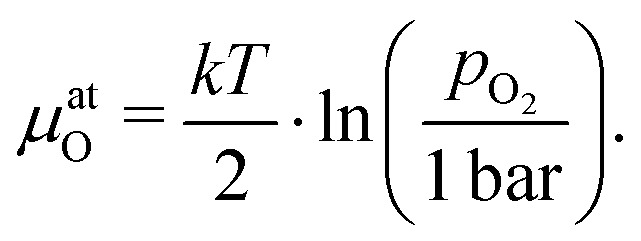



When applying a DC voltage between the working and counter electrode, the resulting chemical potential distribution strongly depends on the spatial position of the rate limiting step. In our case (dense thin film electrodes with current collector grids), we assume the following conditions, in agreement with the literature:^2^,^3^,^7^,^35^

• The electron transport in the electrode film is fast. Thus, electronic transport resistances can be neglected and the electrochemical potential of electrons is constant in the entire electrode film. This is reasonable as LSF has a high electronic conductivity, the across-plane transport length is short (40 nm) and the lateral electron transport is augmented by the current collector.

• The across-plane oxygen transport is fast compared to the surface exchange reaction. Therefore, the entire electrode reaction is surface limited, and the electrochemical potential of oxide ions is constant in the electrode film.

• The YSZ|LSF interface does not contribute any significant transport resistance, therefore no electrochemical potential discontinuity occurs at the interface.

**Fig. 5:**
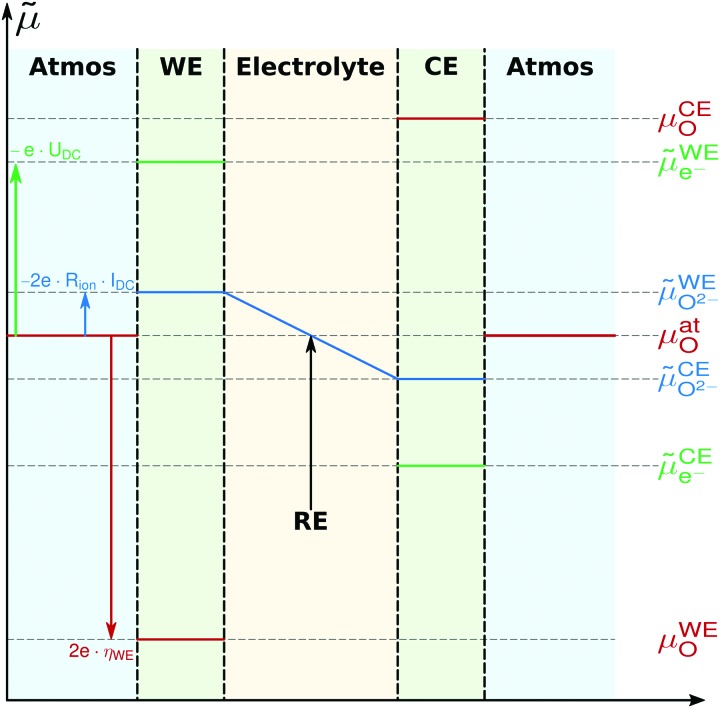
Electrochemical potential distribution of oxygen, oxide ions and electrons in a three-electrode sample during cathodic polarization of the working electrode (WE). Within the electrodes, potentials are constant due to high ionic/electronic conductivity. Potential drops occur at surfaces due to the rate limiting surface exchange reaction. A potential gradient exists in the electrolyte because of finite ionic conductivity. Re and CE denote reference and counter electrode, respectively.

Based on these assumptions, the oxygen chemical potential and the electrochemical potentials of oxide ions and electrons are distributed as depicted in Fig. 5. A current flow *I*_DC_ is imposed between the working and counter electrode, causing a voltage *U*_DC_, *i.e.* an electron electrochemical potential difference between the working and reference electrode. No current flows through the reference electrode, thus it remains in equilibrium with the gas phase, *i.e.* at *μ*REO = *μ*atO. The DC voltage drop

8 *μ̃*WEe– – *μ̃*REe– = –*e*·*U*_DC_

is partially reflected by the oxide electrochemical potential (*μ̃*_O^2–^_) gradient in the electrolyte caused by the ionic transport resistance *R*_ion_. In accordance with Fig. 5, this leads to9
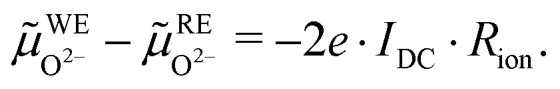
The resulting oxygen chemical potential in the working electrode *μ*WEO, relative to *μ*REO (and thus to *μ*atO), follows from *μ*_O_ = *μ̃*_O^2–^_ – 2*μ̃*_e^–^_. It is given by10
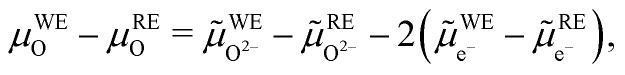
and thus from eqn (8) to (10), we find

11 *μ*WEO – *μ*REO = 2*e*·(*U*_DC_ – *I*_DC_·*R*_ion_) = 2*e*·*η*^WE^,

where *η*^WE^ is the overpotential of the working electrode. The definition of the electrode overpotential explained above (eqn (5)) is in accordance with eqn (11) when neglecting all contributions to *R*_offset_ other than *R*_ion_. Relative to the reference chemical potential of 1 bar oxygen, the oxygen chemical potential inside the working electrode follows from eqn (7) and (11) as12
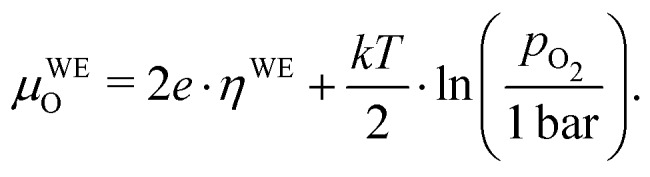
This equation shows that based on the given assumptions, the oxygen chemical potential, and thus the defect chemical state of the working electrode, is unambiguously defined by the oxygen partial pressure in the gas and the electrode overpotential. This equivalence was also used by Kawada *et al.* when analyzing the oxygen vacancy concentration in LSC.^5^

Another point of view on the oxygen chemical potential can be introduced by defining an equivalent oxygen pressure of the working electrode 
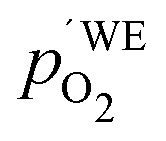
.^5^ This is related to the oxygen chemical potential in the working electrode by13
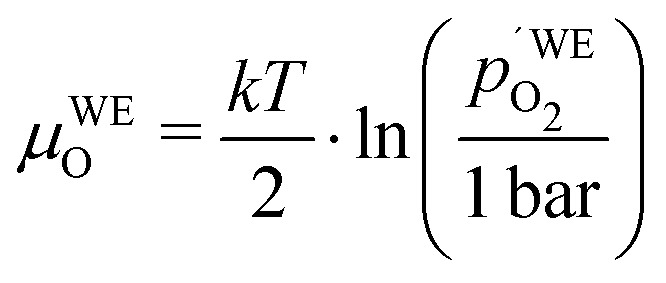
and according to eqn (12), it is thus defined by14
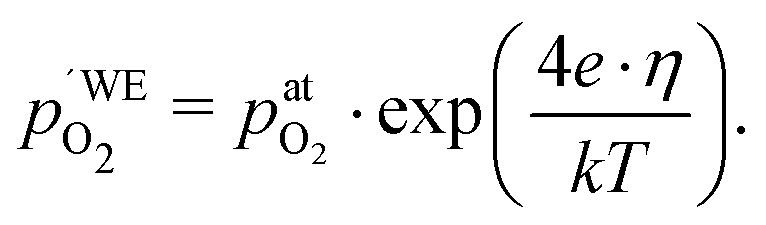



**Fig. 6:**
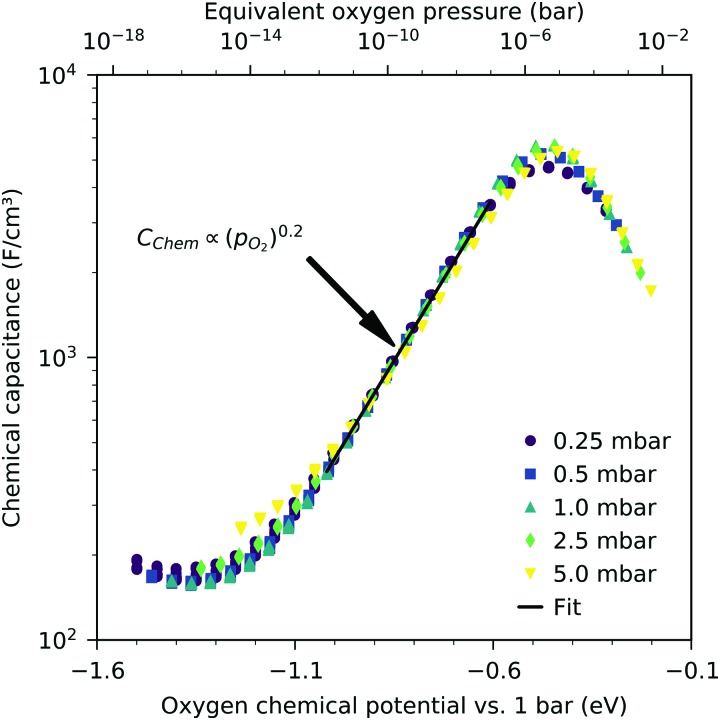
Measured chemical capacitance of a 40 nm thin LSF film at 600 °C (symbols) as a function of the oxygen chemical potential (or equivalent oxygen pressure) of LSF and fit of the values between –1.0 eV and –0.6 eV to a power law (line).

We may now plot the chemical capacitance *versus* the electrode oxygen chemical potential (relative to 1 bar oxygen), *i.e. versus* the equivalent oxygen pressure. This leads to a perfect match of all curves measured in different atmospheres, as shown in Fig. 6. We can thus conclude that the measured capacitance of the LSF films is solely defined by the chemical potential of oxygen within the thin film, regardless of the actual atmospheric oxygen partial pressure or electrode overpotential. This also strongly supports the assumptions made above, particularly the absence of a chemical potential gradient across the LSF film.

The interpretation of the CPE in the impedance analysis in terms of a chemical capacitance is thus reasonable and reflects the defect chemical state of LSF. In the regimes between 2.5 × 10^–4^ bar and 2.0 × 10^–1^ bar oxygen (Fig. 3) and from 10^–7^ to 10^–12^ bar equivalent oxygen pressure (Fig. 6), the *C*_chem_ curves can be fitted to a power law, with exponents of –0.21 (Fig. 3) and 0.20 (Fig. 6), respectively.

### 3.3 Influence of film thickness

**Fig. 7:**
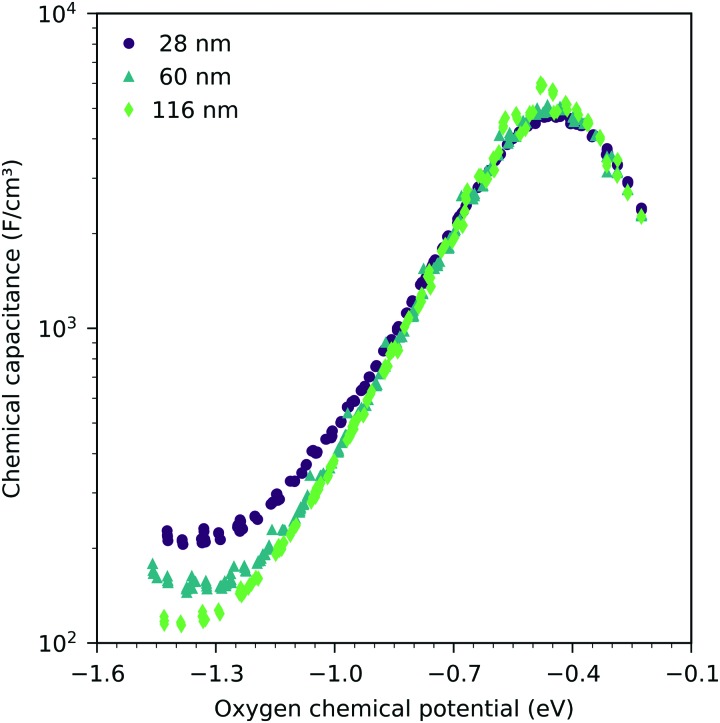
Measured chemical capacitance at 600 °C as a function of the oxygen chemical potential and film thickness. Each curve consists of measurements in different oxygen partial pressures between 2.5 × 10^–4^ bar and 2.5 × 10^–3^ bar oxygen.

Fig. 7 shows the chemical capacitances measured on the LSF films of different thickness, normalized to the LSF film volume. In the regime between –0.1 eV and –0.8 eV oxygen chemical potential, these curves show almost no difference. This means that the area specific capacitances scale linearly with the thin film thickness. This is consistent with the interpretation of this capacitance as the chemical capacitance of the LSF electrode bulk. However, below –0.8 eV, the volume-specific capacitance is higher for the thinner films. These deviations are most prominent around the capacitance minimum at –1.4 eV.

**Fig. 8:**
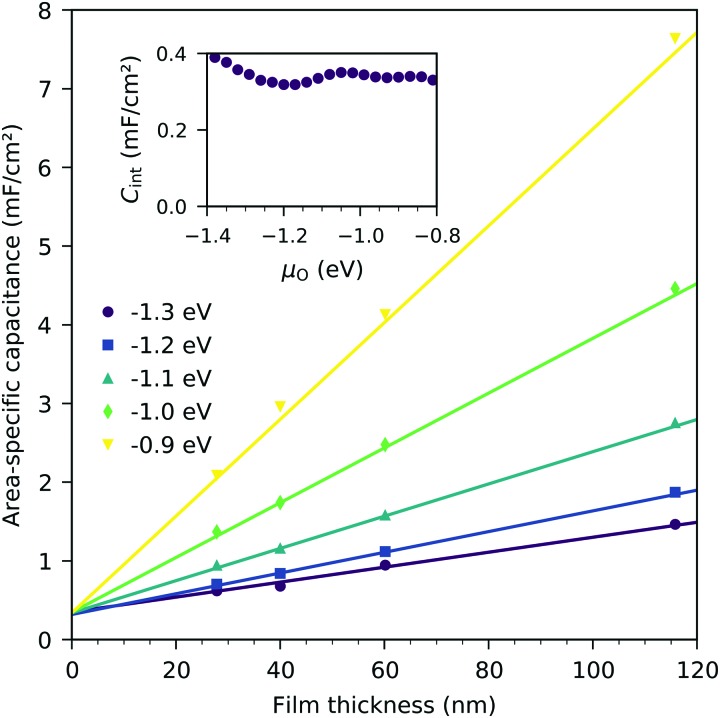
Area-specific capacitance of LSF as a function of film thickness for various oxygen chemical potentials (*μ*_O_), measured at 600 °C. Only the area of LSF above YSZ is considered. Symbols are measured data, and lines represent linear fits. The inset shows the interface capacitance obtained from extrapolating these fits to zero thickness as a function of oxygen chemical potential.

Fig. 8 displays the measured chemical capacitance, normalized to the active electrode area, as a function of film thickness at different oxygen chemical potentials. The measured capacitances show a linear dependence on the film thickness, as expected for a bulk chemical capacitance. However, fitting these data to a linear function and extrapolating to zero film thickness yields an intercept on the capacitance axis of 0.3 to 0.45 mF cm^–2^. Thus, the measured capacitance can be separated into a volume-specific chemical capacitance and a volume independent contribution, most likely due to an interface, either LSF|YSZ, LSF|Pt or LSF|air. Grain boundaries as the origin can be excluded because their contribution should again scale with thickness. Similar volume independent contributions to the measured chemical capacitance have also been found on ceria.^31^ At high oxygen chemical potentials, the volume related chemical capacitance is large and masks the volume independent interfacial capacitance. Close to the capacitance minimum, however, the interface-related capacitance contributes 60% (for 28 nm LSF) to 25% (for 116 nm LSF) to the entire electrode capacitance.

**Fig. 9:**
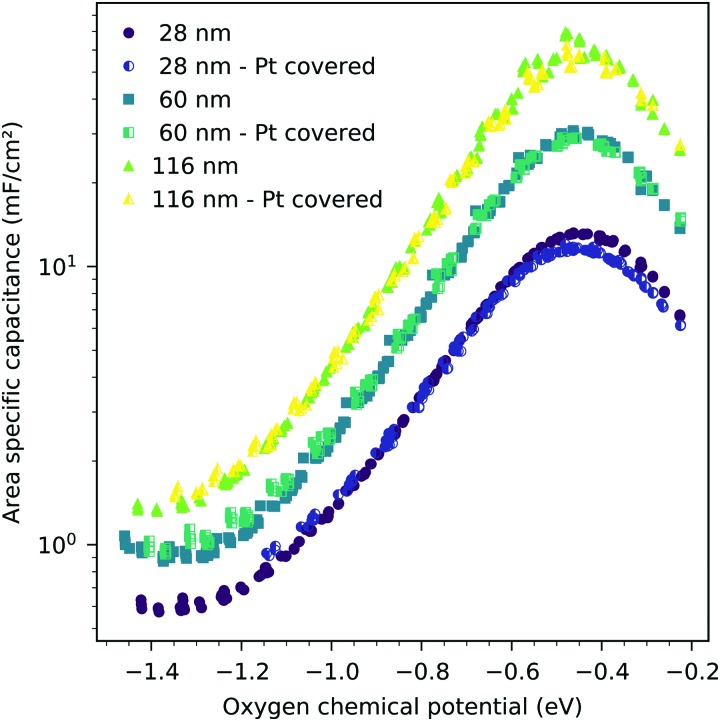
Area-specific capacitance of LSF *versus* oxygen chemical potential for three different film thicknesses, measured at 600 °C, all normalized to the LSF|YSZ interface area. The full symbols are data obtained from the LSF samples without a platinum cover layer, the half filled symbols are data obtained from the LSF samples with a 300 nm platinum layer sputtered onto the LSF surface, eliminating the LSF|air interface.

**Fig. 10:**
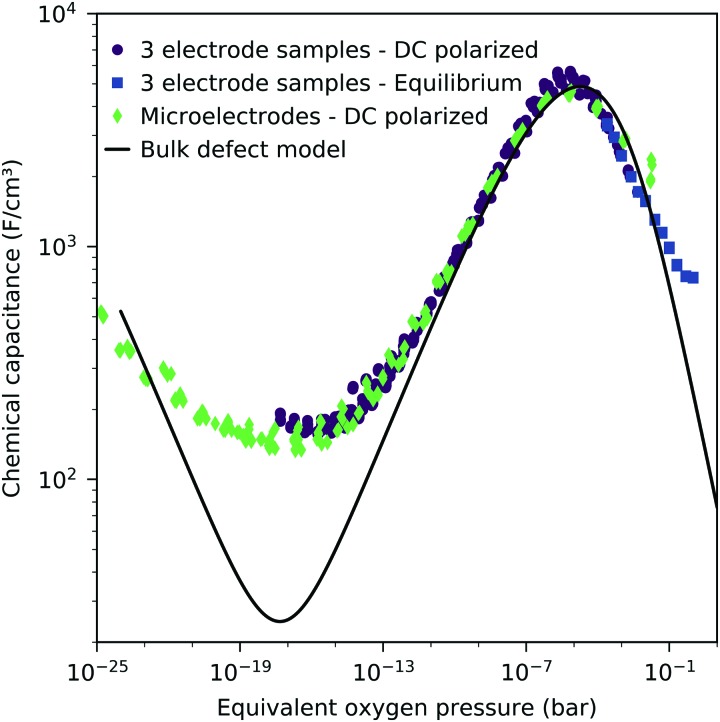
Chemical capacitance at 600 °C as a function of equivalent oxygen partial pressure, measured on the macroscopic three-electrode samples (40 nm) under cathodic bias in 0.25 to 5 mbar oxygen as well as in equilibrium with the gas phase in 0.25 mbar to 1 bar oxygen, and 2-point microelectrode samples (40 nm) in 10 mbar to 1 bar oxygen.

The interfacial capacitance depends only weakly on the oxygen chemical potential, see the inset in Fig. 8, and the measured dependence might easily be an artifact due to the extrapolation for only a few thickness values. To investigate the origin of this capacitance, samples with platinum covered working electrodes were measured. Covering the LSF surface with platinum increases the LSF|Pt interfacial area and eliminates the LSF|air interface. Fig. 9 shows the capacitance measured on the samples with and without platinum on top of LSF as a function of oxygen chemical potential for different film thicknesses. There is almost no difference between the samples with and without the Pt cover, which strongly suggests that the interfacial capacitance is located at the LSF|YSZ interface rather than at the LSF|Pt interface. This conclusion is further supported by a comparison of capacitance measurements on samples with different fractions of the YSZ surface covered by a current collector grid (25% for the three-electrode samples and 75% for the microelectrodes), see Fig. 10. When normalized to the active LSF volume on YSZ, see eqn (4), very good agreement is found. However, which atomistic mechanism at the LSF|YSZ interface causes the corresponding almost *p*_O_2__ independent interfacial capacitance of about 400 μF cm^–2^ remains an open question.

### 3.4 Defect chemical analysis of the chemical capacitance measurements

#### 3.4.1 Bulk defect model

**Fig. 11:**
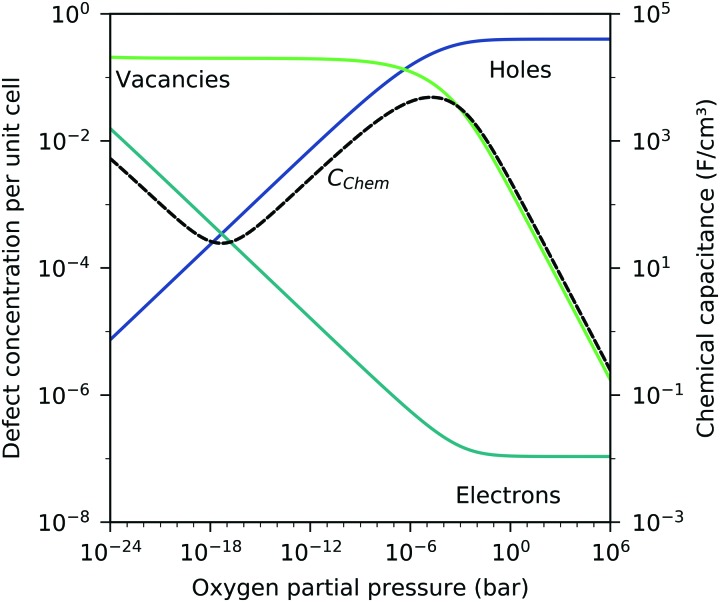
Brouwer diagram for bulk LSF at 600 °C, based on the literature data,^22^ with chemical capacitance calculated according to eqn (22).

For bulk LSF, a defect model was provided by Mizusaki *et al.*^24^,^25^ In this model, the main defect chemical charge carriers are oxygen vacancies 
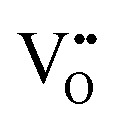
, electrons 
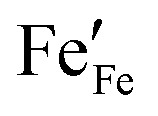
 and electron holes 
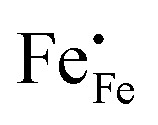
. Enthalpies and entropies for the oxygen incorporation (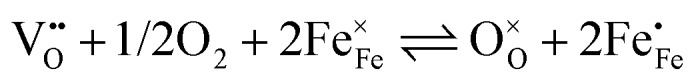) and the electron/hole pair formation (
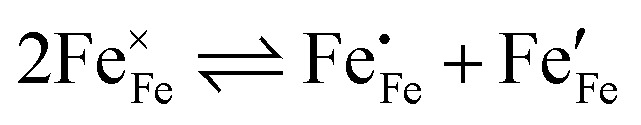
) were determined for bulk LSF by Kuhn *et al. via* thermogravimetry and coulometric titration, see Table 1.^22^ From these data, the defect concentrations can be calculated as a function of oxygen partial pressure; Fig. 11 shows the resulting Brouwer diagram at 600 °C.

For mixed conducting oxides, the chemical capacitance is defined in ref. 30 as15
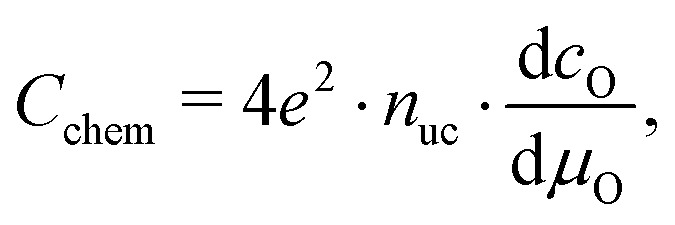
where *c*_O_ denotes the concentration of formally neutral oxygen, *i.e.* the combination of an oxide ion and two electron holes, and *μ*_O_ is the chemical potential of oxygen in the film. The concentration is with respect to one unit cell and *n*_uc_ is the concentration of unit cells. Since for every neutral oxygen atom added, one vacancy is annihilated (d*c*_O_ = –d*c*_V_), we can write16
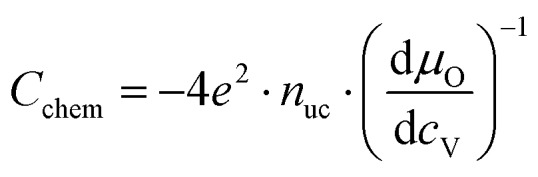
where *c*_V_ is the concentration of oxygen vacancies. The chemical potential of oxygen is

17 *μ*_O_ = *μ*_O^2–^_ + 2*μ*_h_ = –*μ*_V_ + 2*μ*_h_,

where *μ*_O^2–^_, *μ*_V_ and *μ*_h_ denote the chemical potentials of oxide ions, oxygen vacancies and electron holes, respectively. Combining eqn (16) and (17) yields18
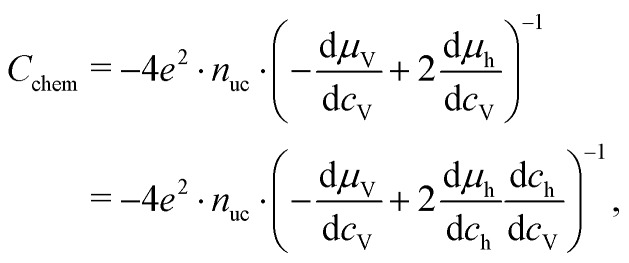
where *c*_h_ is the concentration of electron holes. If the electron concentration is negligibly small, *i.e.* under oxidizing conditions, electroneutrality requires d*c*_h_= –2d*c*_V_ and therefore19
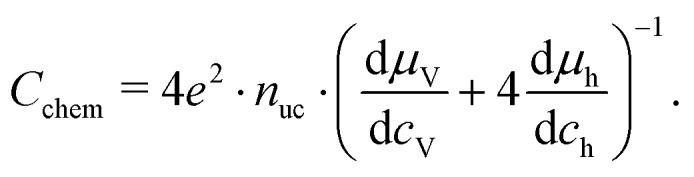
If all defects *i* are sufficiently dilute, their chemical potentials can be described by Boltzmann's statistics with

20 *μ*_*i*_ = *μ*0*i* + *kT* ln (*c*_*i*_),

where *μ*0*i* is the standard chemical potential. We thus obtain21
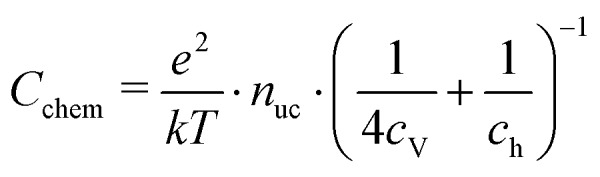
for dilute defects and oxidizing conditions.^29^,^30^,^40^ The same equation with electron concentration instead of hole concentration is valid if electron holes are negligible. For more than two relevant defect species, the equations become more complicated, *cf.*ref. 41. In the case of similar concentrations of dilute electrons and electron holes, we get, for example:22
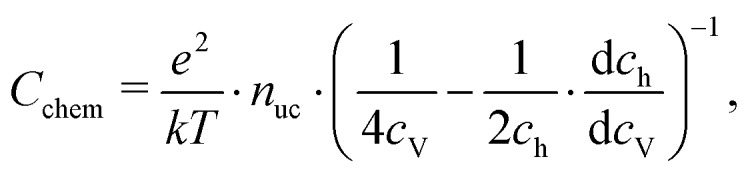
where d*c*_h_ = –2d*c*_V_ + d*c*_e_.

Based on these defect chemical considerations and the thermodynamic data of ref. 22, the volume-specific chemical capacitance of bulk LSF can be calculated and the corresponding curve is given in Fig. 11. At high oxygen partial pressures, the calculated chemical capacitance is determined by the oxygen vacancy concentrations and thus shows a slope of –0.5. Towards lower oxygen partial pressures, it passes a maximum with equally important oxygen vacancies and electron holes. Then, it decreases with decreasing hole concentration (slope 0.25) and reaches a minimum for the electronically intrinsic point (*c*_h_ = *c*_e_). Finally, *C*_chem_ again increases towards even more reducing conditions, in accordance with the increasing electron concentration.

This dilute defect model qualitatively explains the shape of the measured chemical capacitance curve, and in the low *μ*_O_ regime, the exponent of the chemical capacitance *versus* equivalent oxygen partial pressure curve (0.2) is indeed close to the expected value of 0.25. However, two serious deviations become obvious when quantitatively comparing the calculated and measured chemical capacitances, see Fig. 10. At high oxygen partial pressures (above 10 mbar), the measured chemical capacitance decreases in a much shallower manner (–0.21) than predicted by the dilute model (–0.5), and the capacitance value at the minimum is much larger than expected.

The deviation from the ideal behavior for high oxygen partial pressures may be due to hole/hole interactions, since the expected electron hole concentration in this regime is very high (0.4 per unit cell). An excess energy of hole formation due to this interaction might make further oxygen incorporation less favorable, leading to a smaller decrease in the oxygen vacancy concentration, and thus a smaller decrease in chemical capacitance when increasing the oxygen partial pressure. Similar hole/hole interactions were also found for other perovskite materials, such as Ba_1–*x*_La_*x*_FeO_3–*δ*_ and La_1–*x*_Sr_*x*_CoO_3–*δ*_.^42^,^43^ One reason for the increased capacitance around the expected minimum was already identified above and attributed to the LSF|YSZ interface. This interfacial contribution can be subtracted and in the following, the remaining volume-specific chemical capacitance is used to derive thermodynamic data for oxygen incorporation and electron/hole pair formation.

#### 3.4.2 Thermodynamic defect data of LSF films

**Fig. 12:**
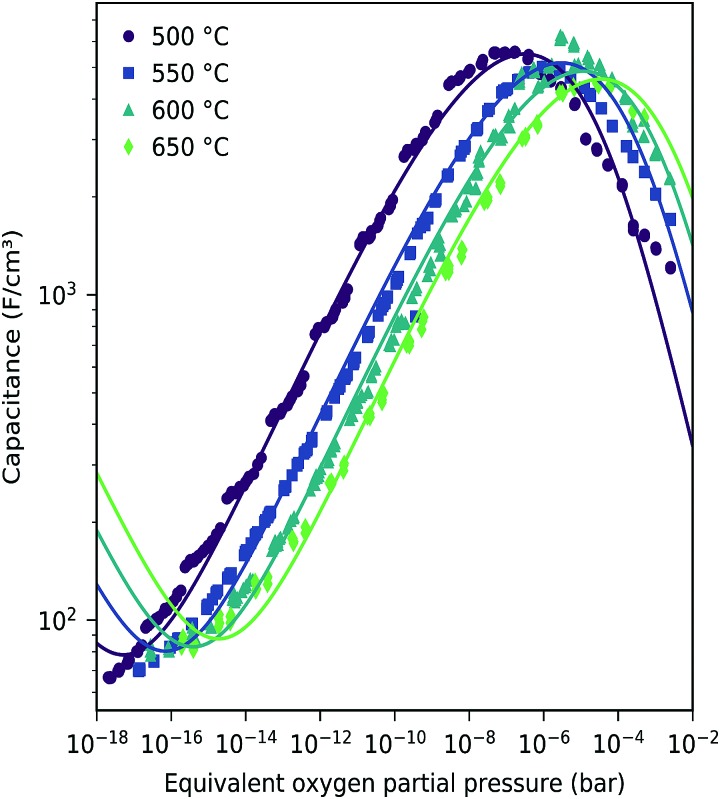
Corrected chemical capacitance of LSF (116 nm thin film) at different temperatures as a function of equivalent oxygen partial pressure. The interface capacitance obtained from film thickness variation at 600 °C (see the inset in Fig. 8) has been subtracted. (Outside the *μ*_O_ range of Fig. 8, the averaged value of *C*_int_ was subtracted.) Symbols are measured data, and lines are fits to the bulk defect model according to eqn (22).

The capacitances of a 116 LSF film were measured at four temperatures between 500 °C and 650 °C, see Fig. 12. Lower temperatures lead to very low relaxation frequencies in the impedance spectra and thus might cause some fit errors, *cf.* the slightly step-like curve at 500 °C. For the sake of simplicity, the curves of all temperatures were corrected by the interfacial capacitance determined at 600 °C, see Fig. 8. Fig. 12 shows the corrected chemical capacitance at different temperatures as a function of oxygen chemical potential.

**Fig. 13:**
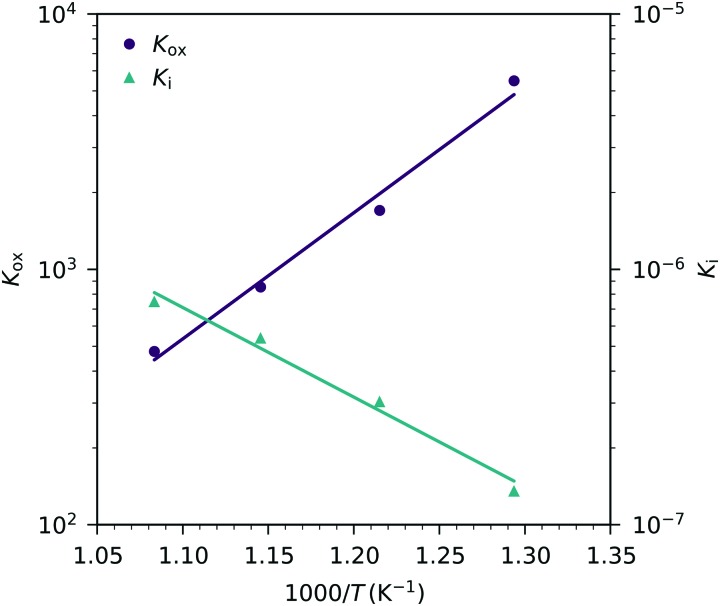
Equilibrium constants for the oxygen incorporation reaction (*K*_ox_) and for the electron/hole pair formation (*K*_i_) obtained from Fig. 12 *versus* inverse temperature and fits according to eqn (25).

These data were fitted based on the dilute bulk defect model and eqn (22) to extract equilibrium constants for the oxygen incorporation reaction23
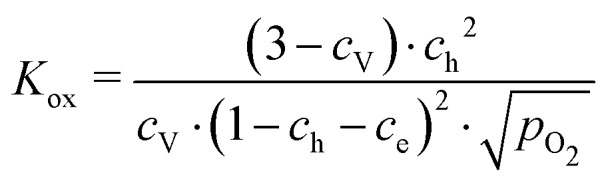
and the electron/hole pair formation24
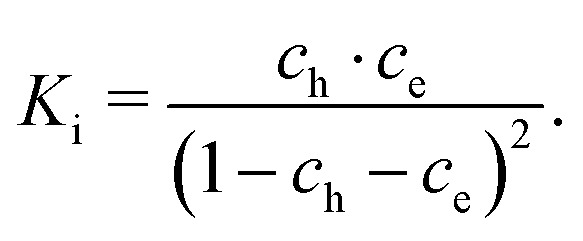
These fits agree very well with our data, with slight deviations in the higher oxygen partial pressure range, probably due to the neglected defect interactions, see above. The equilibrium constants obtained from these fits are shown in the Van't-Hoff plot in Fig. 13. Fitting these to25
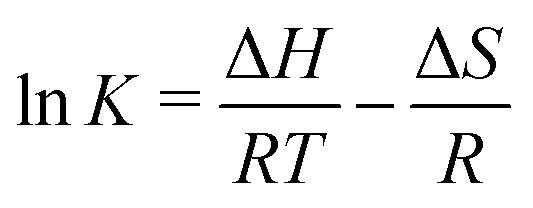
yields enthalpies and entropies for oxygen incorporation and electron/hole pair formation, see Table 1.

**Table 1:** Reaction enthalpies and entropies for oxygen incorporation (Δ*H*_ox_ and Δ*S*_ox_) and electron/hole formation (Δ*H*_i_ and Δ*S*_i_) in LSF extracted from chemical capacitance measurements on thin films, and bulk data from the literature^22^

	This study	Literature (bulk)
Δ*H*_ox_ (kJ mol^–1^)	–94	–95.62 ± 4.18
Δ*S*_ox_ (J mol^–1^ K^–1^)	–50	–54.27 ± 4.43
Δ*H*_i_ (kJ mol^–1^)	26	95.75 ± 2.05
Δ*S*_i_ (J mol^–1^ K^–1^)	–81	–21.63 ± 2.13

The resulting oxygen incorporation entropy and enthalpy agree very well with the thermodynamic data of bulk LSF.^22^ However, the deduced electron/hole pair formation enthalpy and entropy are both lower than those for bulk LSF.^22^,^24^,^25^ Possible reasons include the effects of film strain and its changes with changing oxygen chemical potential, and some differences in the exact stoichiometry (cation vacancies) or different pressure ranges used to deduce data, see below. Additionally, grain boundaries in our nanocrystalline thin films may cause deviations from the literature bulk data. Since the oxidation state in the grain boundaries is probably different than in the grains, a shifted *C*_chem_*vs. p*_O_2__ curve results, which adds to the grain chemical capacitance. A more detailed analysis, however, would require further data points in the very low *p*_O_2__ region beyond the capacitance minimum.

**Fig. 14:**
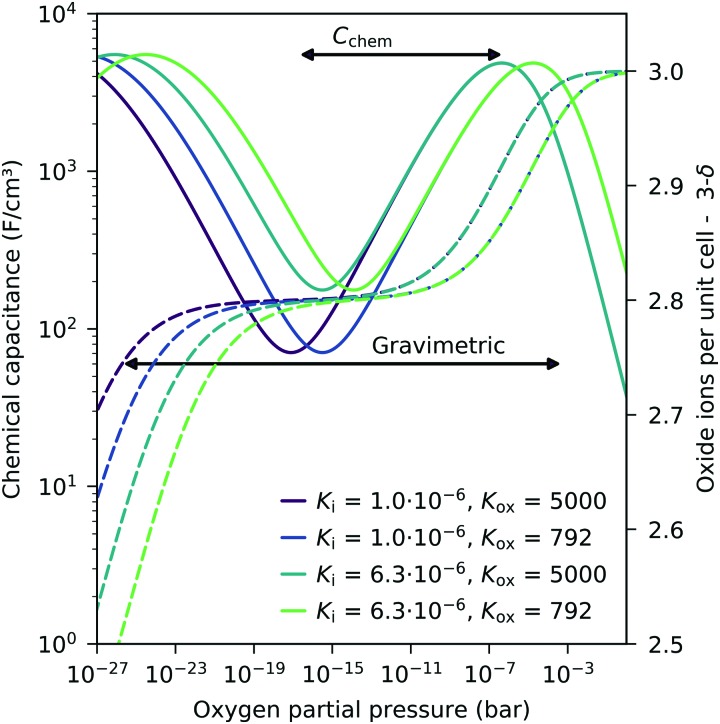
Calculated chemical capacitance for LSF (solid lines) for different equilibrium constants and the corresponding oxygen stoichiometry (dashed lines), both based on the dilute defect model, see eqn (22). A much narrower *p*_O_2__ span (only from capacitance maximum to capacitance minimum) is sufficient to obtain both equilibrium constants by chemical capacitance measurements, compared to thermogravimetric measurements (both steps in the oxide concentration are required). Arrows indicate typical ranges required for a fit analysis of *C*_chem_ or weight analysis.

These experiments demonstrate that chemical capacitance measurements are a powerful alternative method for analyzing the defect concentrations and defect thermodynamics of oxide materials, particular for thin films. Such an approach to the defect chemistry by *C*_chem_ measurements even has some advantages compared to common gravimetric studies. Since the chemical capacitance probes primarily minority charge carriers, it might be more sensitive to defect interactions causing vacancy changes in the hole conducting regime (see above). Moreover, compared to gravimetric studies, a less broad *p*_O_2__ regime is already sufficient to obtain thermodynamic data. This is detailed in Fig. 14 for different equilibrium constants. In gravimetric studies, both steps indicating weight loss for increasing vacancies have to be observed. In *C*_chem_ studies, however, only the regime between the capacitance maximum and minimum is required.

## 4 Conclusions

Dense LSF thin film electrodes were prepared on YSZ electrolyte substrates and their chemical capacitance was measured by impedance spectroscopy in different atmospheres and with varying DC polarizations. These measurements revealed the following:

• The chemical capacitance solely depends on the oxygen chemical potential in the electrode film, independent of the source affecting this potential (atmospheric oxygen pressure or external DC voltage).

• The shape of the chemical capacitance *versus* oxygen chemical potential curve is in qualitative agreement with expectations from bulk defect chemical models and already indicates the relevant minority defects determining the chemical capacitance.

• For very small chemical capacitances, *i.e.* for moderately reducing conditions, an additional interfacial contribution to the capacitance in the range of 400 μF cm^–2^ comes into play and can be attributed to the LSF|YSZ interface.

• The corrected volume-specific chemical capacitance of the thin films can be reasonably well approximated by a defect chemical model with dilute defects, thus mass action constants for the oxygen exchange reaction and the electron/hole formation can be determined.

• The temperature dependence of the determined defect chemical data revealed the enthalpy and entropy of the oxygen incorporation reaction and of the electron–hole formation.

• Oxygen incorporation enthalpies and entropies of the LSF films agree very well with the literature data obtained on macroscopic samples. Electronic defect formation enthalpies and entropies, however, differ, leading to higher electronic defect concentrations in the thin films. Moreover, defect interactions between electron holes may play a role at high oxygen partial pressures, leading to deviations from the *C*_chem_ values predicted by the dilute defect model.

• The measurements showed that bias and oxygen partial pressure dependent chemical capacitance measurements can be a powerful tool for the analysis of the defect chemistry of thin films, and particularly to reveal details of the minority defects.

## Conflicts of interest

There are no conflicts of interest to declare.
